# 血小板输注对^60^Co照射所致小鼠骨髓造血龛损伤修复和骨髓移植后造血重建的影响

**DOI:** 10.3760/cma.j.issn.0253-2727.2023.08.004

**Published:** 2023-08

**Authors:** 云 刘, 爽 丁, 静芳 孙, 朋朋 李, 晓倩 李, 令宇 曾, 开林 徐, 建林 乔

**Affiliations:** 1 徐州医科大学附属医院检验科，徐州 221000 Department of Clinical Laboratory, the Affiliated Hospital of Xuzhou Medical College, Xuzhou 221002, China; 2 徐州医科大学血液病研究所，江苏省骨髓干细胞重点实验室，徐州医科大学附属医院血液科，徐州 221000 Blood Disease Institute, Xuzhou Medical University, Key Laboratory of Bone Marrow Stem Cell, Department of Hematology, the Affiliated Hospital of Xuzhou Medical University, Xuzhou 221000, China

**Keywords:** 血小板输注, 骨髓移植, 小鼠, 造血微环境, Platelet transfusion, Bone marrow transplantation, Mouse, Hematopoietic microenvironment

## Abstract

**目的:**

观察血小板输注对^60^Co照射所致小鼠骨髓造血龛损伤修复和骨髓移植后造血重建的影响。

**方法:**

①以8～10周雄性C57BL/6小鼠为研究对象，接受^60^Co全身照射后分为单独照射组和照射输注组（经尾静脉输注1×10^8^个表达GFP荧光蛋白的血小板）。②建立小鼠异基因造血干细胞移植模型，分为单纯移植组（BMT组）和移植+血小板输注组（BMT+PLT组）。BMT组仅通过尾静脉输注5×10^6^个骨髓细胞，BMT+PLT组在输注骨髓细胞的同时输注1×10^8^个血小板。检测外周血血细胞和骨髓细胞计数，流式细胞术检测骨髓造血干细胞（HSC）和造血祖细胞（HPC）比例、骨髓细胞增殖率和凋亡率，病理观察造血龛的损伤与修复。

**结果:**

①在照射后第3、7、14和21天，照射输注组骨髓细胞计数和外周血细胞计数均高于单纯照射组（*P*值均<0.05），第21天输注组白细胞计数高于单纯照射组，第7天血小板计数高于单纯照射组（*P*值均<0.05）。在观察周期内照射输注组骨髓细胞增殖率较高，而凋亡率较低；②免疫荧光结果显示照射输注组造血龛的连续性更好；③在移植后，BMT+PLT组嵌合率始终高于BMT组；④BMT+PLT组在移植后第7、28天的骨髓细胞计数和骨髓细胞增殖率均高于BMT组，第14天骨髓细胞凋亡率低于BMT组（*P*值均<0.05），骨髓细胞中HSC、HPC占比在第14天以后均高于BMT组（*P*值均<0.05）；⑤骨髓组织的免疫组化染色结果显示BMT+PLT组血管内皮连续性更好。结论 输注血小板可减轻^60^Co照射所致小鼠骨髓造血龛的损伤，有利于骨髓移植后造血重建。

## Abstract (en)

**Objective:**

To observe the effect of platelets on hematopoietic stem cell（HSCs）implantation in mice with radiation-induced bone marrow injury and bone marrow transplantation models.

**Methods:**

① Male C57BL/6 mice were divided into a single irradiation group and a radiation infusion group after receiving ^60^Co semimyeloablative irradiation for 18–10 weeks. The irradiation infusion group received 1×10^8^ platelets expressing GFP fluorescent protein. ② The allogeneic bone marrow transplantation model was established. The experimental groups included the simple transplantation group（BMT）and the transplantation infusion group（BMT+PLT）. The BMT group was infused through the tail vein only 5 × 10^6^ bone marrow cells, the BMT+PLT group needs to be infused with bone marrow cells at the same time 1× 10^8^ platelets. ③ Test indicators included peripheral blood cell and bone marrow cell counts, flow cytometry to detect the proportion of hematopoietic stem cell（HSC）and hematopoietic progenitor cells, bone marrow cell proliferation and apoptosis, and pathological observation of vascular niche damage and repair.

**Results:**

① On the 3rd, 7th, 14^th^, and 21st days after irradiation, the bone marrow cell count of the infusion group was higher than that in the single irradiation group（*P*<0.05）, and the peripheral blood cell count was also higher. A statistically significant difference was found between the white blood cell count on the 21st day and the platelet count on the 7th day（*P*<0.05）. In the observation cycle, the percentage of bone marrow cell proliferation in the infusion group was higher, while the percentage of apoptosis was lower. ② The results of bone tissue immunofluorescence after irradiation showed that the continuity of hematopoietic niche with red fluorescence was better in the irradiation infusion group. ③ The chimerism percentage in the BMT+PLT group was always higher than that in the BMT group after transplantation.④ The BMT+PLT group had higher bone marrow cell count and percentage of bone marrow cell proliferation on the 7th and 28th day after transplantation than that in the BMT group, and the percentage of bone marrow cell apoptosis on the 14th day was lower than that in the BMT group（*P*<0.05）. After the 14th day, the percentage of stem progenitor cells in the bone marrow cells of mice was higher than that in the BMT group（*P*<0.05）. ⑤ The immunohistochemical results of bone marrow tissue showed that the continuity of vascular endothelium in the BMT+PLT group was better than that in the BMT group.

**Conclusion:**

Platelet transfusion can alleviate the injury of vascular niche, promotes HSC homing, and is beneficial to hematopoietic reconstruction.

造血干细胞移植是治疗恶性血液系统疾病的有效手段，移植前的全身放射治疗（TBI）以及化疗在消灭肿瘤细胞、抑制患者的免疫功能的同时不可避免地造成全身广泛的内皮损伤，从而产生造血干细胞（hematopoietic stem cell, HSC）植入障碍、造血延迟等移植后并发症，严重影响了移植患者的生存质量。骨髓造血微环境又称造血龛（hematopoietic niche），是HSC赖以生存的场所，其完整性是保证HSC有效植入发挥造血功能的首要条件[1]。TBI使造血龛严重受损，影响HSC的定植以及造血功能的发挥，寻求有效的治疗策略是解决此问题的关键[2]。

研究表明在组织受损时，血小板最先到达受损部位，发生活化后向微环境中释放生物活性物质参与组织修复[3]。血小板及其制剂被越来越多地用于治疗糖尿病足以及皮肤、视网膜、角膜、鼓膜、软骨损伤[4]–[7]。以往研究证明在血管发生损伤时，活化血小板黏附在内皮细胞上，释放基质细胞衍生因子-1（SDF-1）、血小板因子4（PF4）、血管内皮生长因子（VEGF）、血小板衍生生长因子（PDGF）、转化生长因子-β（TGF-β）、表皮生长因子（EGF）、胰岛素样生长因子（IGF）、血管生成素相关生长因子（AGF）等介质和促血管生成因子来参与组织修复[8]–[10]。本研究通过建立骨髓造血龛损伤模型和小鼠骨髓移植模型，探索血小板在骨髓造血龛修复中的作用及对HSC植入的影响，为寻求新的加速移植后HSC植入和受损造血龛修复的策略提供新思路。

## 材料与方法

一、实验动物

8～10周龄C57BL/6小鼠用于建立照射致骨髓造血龛损伤模型，4～6周龄C57BL/6带GFP荧光小鼠用于制备洗涤血小板，6～8周龄BALB/c（H-2K^d^）小鼠做为移植受鼠，8～10周龄C57BL/6（H-2K^b^）小鼠作为移植供鼠和制备洗涤血小板。以上小鼠均为无特定病原体（SPF）级雄性小鼠，体重20～28 g，购于上海斯莱克实验动物技术有限公司，饲养于徐州医科大学实验动物中心恒温恒湿屏障系统。

二、主要试剂与仪器

Anti-GFP antibody（碧云天生物技术研究所）；lin系抗体（CD45R/B220-FITC、Mac-1-FITC、Gr-1-FITC、CD4-FITC、CD8a-FITC、Tre119-FITC）、anti-mouse c-kit-PE、anti-mouse Sca-1-APC（美国BD公司）；anti-mouse Ki-67-FITC、anti-mouse Annexin V-FITC（美国Biolegend公司）；Anti-mouse VE Cadherin antibody（英国Abcam公司）；^60^Co治疗机（山东新华医疗器械公司）；石蜡切片机和冰冻切片机（德国Leica公司）；FACS Calibur流式细胞仪（美国BD公司）；倒置显微镜IX71（日本OLYMPUS公司）；血细胞计数仪（深圳迈瑞生物医疗公司）。

三、血小板输注对骨髓照射损伤小鼠造血重建的影响

1. 实验小鼠照射前处理：将SPF级8～10周雄性C57BL/6小鼠转移至层流架中，于照射预处理前1周饮用含庆大霉素（32万U/L）和头孢曲松钠（0.25 g/L）的灭菌水及喂食高压后的大米燕麦等软食。

2. 用GFP荧光小鼠制备洗涤GFP-PLT：在超净工作台中用10％水合氯醛麻醉小鼠后摘眼球取血（柠檬酸葡萄糖抗凝剂抗凝），在全血中加入含胎牛血清蛋白（BSA）的血小板洗涤缓冲液（PWB）300 µl颠倒混匀，×250 *g*离心2 min，将离心后的上清液转移至新的Ep管中，重复以上步骤直至肉眼观察上清液中没有红细胞，×2 000 *g*离心1 min后弃上清液，在沉淀中加入1 ml PWB（不含BSA）重悬混匀，用血细胞计数仪行血小板计数，随后×2 000 *g*离心1 min，弃上清液，用无菌生理盐水重悬，制成4×10^8^个/ml浓度的血小板悬液置于20～24 °C震荡保存箱。

3. 实验分组：将8～10周雄性C57BL/6小鼠随机分为3组：①正常对照组（*n*＝6）：不接受全身照射，仅尾静脉输注0.25 ml生理盐水；②单纯照射组（*n*＝25）：给予^60^Co单次全身照射，总剂量5.5 Gy，剂量率0.67 Gy/min，并于照射后4 h尾静脉输注0.25 ml生理盐水；③照射+血小板输注组（*n*＝25）：同剂量照射后4 h经尾静脉输注0.25 ml GFP-PLT。

4. 检测指标

（1）血细胞计数：在处理后第3、7、14、21、28天，采用10％水合氯醛麻醉小鼠，摘眼球取血（EDTA抗凝）用于血细胞计数。

（2）骨髓细胞计数：将小鼠脱颈处死后取两侧胫骨、髂骨，剪开骨干两端，用磷酸盐缓冲液（PBS）冲洗骨髓腔及干骺端，随后用200目滤网过滤后收集骨髓细胞悬液置于离心管内，用×400 *g*离心2 min，弃上清后将沉淀用PBS定容至1 ml，取10 µl混悬液用2.5％醋酸稀释后计数骨髓细胞总数。

（3）流式细胞术检测骨髓HSC、造血祖细胞（HPC）、骨髓细胞增殖率及凋亡率：收集小鼠左侧股骨骨髓细胞悬液，置于离心管内备用：①骨髓HSC（CD45^−/low^ c-kit^+^ Lin^−^ Sca-1^+^）和HPC（CD45^−/low^ c-kit^+^ Lin^−^Sca-1^−^）检测：在流式上机管中，分别加入相应抗体各2 µl、骨髓细胞悬液50 µl、PBS 50 µl，避光孵育20 min，加入红细胞裂解液600 µl裂解5 min，PBS洗涤1次，×400 *g*离心2 min，200 µl PBS重悬细胞，上流式细胞仪检测。②骨髓细胞增殖率检测：在流式上机管中，分别加入抗体c-kit-PE 2 µl、Sca-1-APC 2 µl、骨髓细胞悬液50 µl、PBS 50 µl，避光孵育20 min，各管加入经−20 °C预冷的70％乙醇3 ml，并置于−20 °C冰箱1 h。×350 *g* 4 °C离心5 min后弃去上清，加入1 ml 染色缓冲液洗涤2次，各管加入2 µl抗体 Ki-67-FITC，室温避光孵育30 min后加入1 ml 染色缓冲液洗涤，以200 µl PBS重悬细胞，上流式细胞仪检测。③骨髓细胞凋亡率检测：在流式上机管中，分别加入抗体AnnexinⅤ 2 µl、骨髓细胞悬液50 µl、染色缓冲液50 µl，避光孵育15 min后，×350 *g* 4 °C离心5 min，弃上清，以300 µl染色缓冲液重悬后，各管加入5 µl 7-氨基放线菌素D（7-AAD），上流式细胞仪检测。

（4）免疫荧光观察小鼠股骨组织荧光表达：取小鼠右侧股骨，以及小鼠肝脏、肺脏、脾脏，置于4％多聚甲醛中固定48 h，脱水后包埋各组织，制作冰冻切片，经0.5％ TritonX-100室温孵育15 min后用PBS洗涤，用山羊血清封闭组织1 h后滴加GFP荧光抗体，并于4 °C避光孵育过夜，次日平衡室温后用滴加4′，6-二脒基-2-苯基吲哚（DAPI）孵育15 min后，用PBS洗涤，最后用防淬灭剂封片，于荧光显微镜下观察骨髓组织中荧光表达情况。

（5）免疫组化染色观察小鼠股骨组织造血龛的损伤：向用山羊血清封闭1 h后的股骨组织冰冻切片中滴加抗血管内皮钙粘附素（VE-Cadherin）的抗体，4 °C避光孵育过夜，次日平衡室温后加入二抗避光孵育1 h，滴加DAPI孵育15 min后，用PBS洗涤，防淬灭剂封片后用共聚焦显微镜观察。

四、血小板输注对骨髓移植小鼠造血重建的影响

1. 实验分组：照射前小鼠处理和洗涤血小板制备同上。①正常对照组（*n*＝6）：仅尾静脉输注PBS 0.25 ml；②单纯移植组（BMT组，*n*＝25）：单次给予^60^Co全身照射，总剂量7.5 Gy，剂量率0.67 Gy/min，照射后4 h，每只小鼠尾静脉输注骨髓单个核细胞5×10^6^个（0.25 ml）；③移植联合血小板输注组（BMT+PLT组，*n*＝25）：照射后4 h，给予尾静脉输注骨髓单个核细胞5×10^6^个（0.15 ml），尾静脉注射血小板悬液1×10^8^（0.1 ml）。第7、14、21、28天同法处理小鼠。

2. 流式细胞术检测骨髓细胞嵌合率：流式上机管中加入anti-CD45-PerCPCy5.5抗体2 µl、anti-H-2K^b^-PE抗体2 µl、anti-H-2K^d^-FITC抗体2 µl、骨髓细胞悬液50 µl、PBS 50 µl，避光孵育20 min，以红细胞裂解液600 µl裂解红细胞5 min，PBS洗涤1次，×350 *g*离心2 min，200 µl PBS重悬细胞，流式细胞术检测嵌合率。

3. 免疫组化染色：取移植后小鼠股骨，4％多聚甲醛固定48 h，经脱钙、脱水、浸蜡，5 µm切片后行免疫组化染色。

五、统计学处理

采用SPSS21.0和GraphPadPrism5.0进行统计学分析和绘图，符合正态分布的计量资料用“均数±标准差（*x±s*）”表示；两组间比较若方差齐采用*t*检验，方差不齐采用Mann-Whitney非参数检验，*P*<0.05表示差异有统计学意义。

## 结果

一、血小板输注对^60^Co照射小鼠造血重建的影响

在照射后第3、7、14、21天，照射输注组骨髓细胞计数均高于单纯照射组（*P*<0.05），在第28天两组差异无统计学意义（表1）。

**表1:** 照射后各指标随时间的变化（*n*＝6, *x±s*）

检测指标及时间（d）	单纯照射组	照射输注组	*t*值	*P*值
BMC（×10^7^/L）				
3	0.59±0.08	1.06±0.14	−4.037	0.027
7	0.24±0.03	0.39±0.03	−6.769	0.002
14	1.20±0.43	2.45±0.90	−2.493	0.047
21	2.50±0.46	5.64±1.58	−3.319	0.029
28	7.40±0.42	6.80±0.16	2.257	0.109
WBC（×10^9^/L）				
3	0.45±0.04	0.28±0.09	2.310	0.104
7	0.31±0.21	0.17±0.06	1.233	0.264
14	0.28±0.08	0.30±0.12	−0.306	0.770
21	2.35±0.55	3.87±0.74	−2.863	0.046
28	3.08±1.37	2.46±0.11	2.261	0.152
RBC（×10^12^/L）				
3	8.03±0.58	9.20±0.40	−2.871	0.052
7	7.38±0.57	8.35±0.98	−1.714	0.137
14	5.38±0.99	5.07±0.97	0.448	0.670
21	7.93±1.06	8.05±1.00	−0.193	0.851
28	6.34±0.68	7.26±0.57	−1.778	0.150
PLT（×10^9^/L）				
3	1022.5±23.33	1014.0±65.02	0.170	0.876
7	261.25±27.68	327.25±41.16	−2.661	0.037
14	119.25±63.09	159.50±35.18	−1.114	0.308
21	755.80±113.5	842.30±175.2	−1.015	0.334
28	943.5±116.67	820.30±103.37	1.249	0.300
HSC（%）				
3	0.63±0.52	2.17±1.43	−1.432	0.289
7	1.30±0.40	1.30±0.35	0.000	1.000
14	9.93±2.61	8.55±2.64	0.689	0.522
21	0.82±0.58	1.00±0.62	−0.381	0.723
28	2.50±1.50	2.38±0.21	0.141	0.895
HPC（%）				
3	4.25±2.69	9.66±2.61	−2.250	0.110
7	3.25±1.01	4.20±1.05	−1.207	0.282
14	20.83±6.43	23.43±1.38	−0.839	0.429
21	5.60±1.91	5.95±0.78	−0.240	0.833
28	16.62±2.40	15.74±1.54	1.158	0.311
骨髓细胞增殖率（%）				
3	2.89±0.81	3.88±0.67	−1.636	0.177
7	20.98±1.82	25.50±3.12	−2.586	0.042
14	24.59±2.97	26.22±5.95	−0.714	0.496
21	2.02±0.59	6.05±1.22	−4.187	0.025
28	2.32±1.14	5.59±0.93	−3.344	0.044
骨髓细胞凋亡率（%）				
3	1.53±0.76	1.56±0.29	0.767	0.919
7	3.60±0.90	2.08±0.33	3.199	0.024
14	6.48±1.08	5.65±1.10	1.134	0.294
21	4.23±0.03	2.69±0.25	8.400	0.004
28	4.45±1.24	4.34±0.30	0.122	0.914

**注** BMC：骨髓细胞；HSC：造血干细胞；HPC：造血祖细胞

照射输注组WBC在照射后第21天高于单纯照射组［（3.87±0.74）×10^9^/L对（2.35±0.55）×10^9^/L，*P*＝0.046］，血小板计数在第7天高于单纯照射组［（327.25±41.16）×10^9^/L对（261.25±27.68）×10^9^/L，*P*＝0.037］。

照射输注组与单纯照射组相比，骨髓细胞增殖率更高，而凋亡率更低。在第7、21、28天两组骨髓细胞增殖率差异有统计学意义，第7、21天两组骨髓细胞凋亡率差异有统计学意义（表1）。

二、共聚焦显微镜观察^60^Co照射后骨髓造血龛的损伤情况

对照射后第3天的小鼠骨髓组织进行免疫荧光染色，在共聚焦显微镜下可以看到照射输注组造血龛（呈现红色荧光）的连续性优于单纯照射组（图1）。

**图1:**
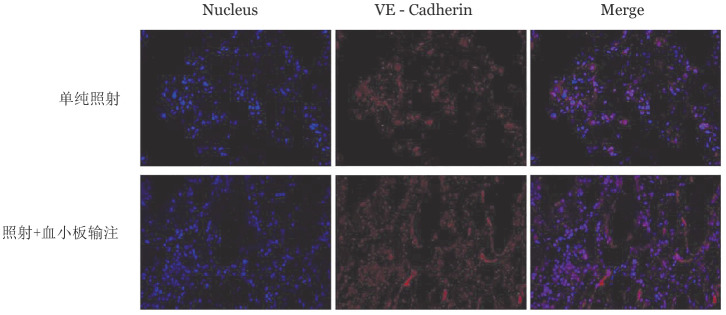
血小板输注对小鼠^60^Co照射后骨髓造血龛修复的影响（免疫荧光染色，×400）

三、骨髓移植后嵌合率的变化

骨髓移植后BMT+PLT组嵌合率始终高于BMT组。在第7天两组差异有统计学意义［（64.79±5.15）％对（56.46±5.67）％，*P*＝0.031］。详见表2。

**表2:** 血小板输注对小鼠异基因骨髓移植后骨髓细胞嵌合率的影响（％, *x±s*, *n*＝6）

组别	第7天	第14天	第21天	第28天
BMT组	56.46±5.67	88.93±2.91	91.60±2.95	92.83±3.66
BMT+PLT组	64.79±5.15	89.53±2.78	91.54±1.53	95.29±1.01

*t*值	−2.552	−0.345	−0.062	−1.287
*P*值	0.031	0.738	0.952	0.234

**注** BMT组：单纯骨髓移植组；BMT+PLT组：骨髓移植联合血小板输注组

四、移植后小鼠造血重建情况

移植后在第7天和28天BMT+PLT组骨髓细胞计数和骨髓细胞增殖率均高于BMT组，且差异具有统计学意义。第14天BMT+PLT组骨髓细胞的凋亡百分比低于BMT组。小鼠骨髓细胞中干祖细胞百分比在第14天以后高于BMT组，差异具有统计学意义。详见表3。

**表3:** 血小板输注对移植后小鼠各项指标的影响（*n*＝6, *x±s*）

检测指标及时间（d）	BMT组	BMT+PLT组	*t*值	*P*值
BMC（×10^7^/L）				
7	1.67±0.42	2.56±0.53	−2.669	0.037
14	5.76±1.13	4.88±0.91	1.494	0.166
21	3.63±0.99	4.01±1.00	−0.718	0.489
28	4.21±0.96	6.21±1.32	−2.607	0.035
HSC（％）				
7	1.11±0.52	0.55±0.20	2.251	0.055
14	9.42±2.09	17.87±2.10	−6.979	0.000
21	3.83±1.21	7.26±1.47	−4.419	0.001
28	2.74±1.03	7.87±3.02	−3.923	0.004
HPC（％）				
7	2.64±0.72	1.81±0.47	2.102	0.074
14	4.93±1.13	6.65±0.62	−2.678	0.037
21	2.24±0.33	3.33±0.52	−3.217	0.018
28	9.27±1.90	29.58±12.22	−3.705	0.021
骨髓细胞增殖率（％）				
7	20.17±10.67	38.75±15.31	−2.282	0.048
14	11.37±0.55	11.45±2.57	−0.078	0.940
21	26.62±6.08	33.83±6.80	−1.767	0.115
28	7.37±1.32	13.24±3.87	−3.242	0.018
骨髓细胞凋亡率（％）				
7	0.31±0.12	0.36±0.15	−0.650	0.530
14	2.13±0.25	1.64±0.08	3.176	0.034
21	2.83±0.42	3.13±0.89	−0.764	0.463
28	3.93±0.49	3.47±0.39	1.500	0.177

**注** BMC：骨髓细胞；HSC：造血干细胞；HPC：造血祖细胞

五、免疫组化染色观察骨髓造血龛的受损情况

正常对照组骨髓造血龛见图2。在骨髓移植后第7天，BMT组和BMT+PLT组骨髓造血龛均受损严重，随着时间推移，第14和21天的骨髓组织免疫组化结果显示BMT+PLT组比BMT组造血龛的连续性更好，损伤情况更轻，直至恢复正常结构（图3）。

**图2:**
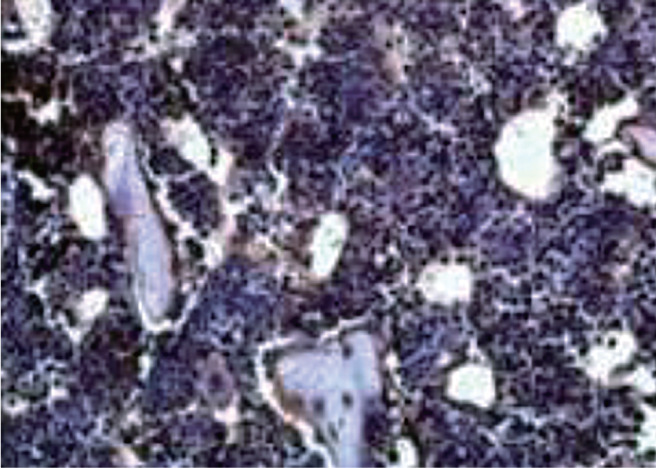
免疫组化染色观察正常对照小鼠骨髓造血龛（×400）

**图3:**
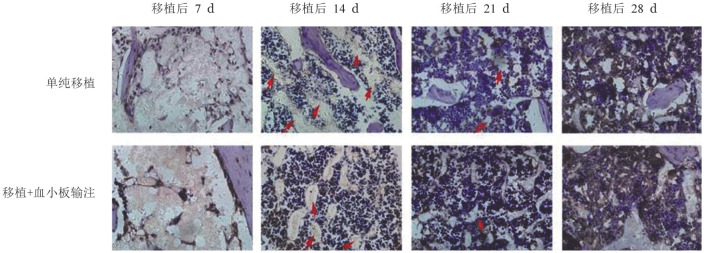
免疫组化染色观察血小板输注对小鼠骨髓移植后造血龛修复的影响（×400，红色箭头示造血龛缺损部位）

## 讨论

随着对血小板功能研究的不断深入，研究发现活化后的血小板通过表达黏附分子和脱颗粒参与到人体的病理生理过程当中[11]。血小板在血管损伤后可到达受损部位，参与受损血管的修复，主要机制包括：释放各种细胞因子、趋化因子和生长因子促进细胞迁移和增殖，释放大量与血管生成和抗血管生成有关介质促进血管生成，释放促凋亡和抗凋亡介质控制细胞凋亡/存活以及释放一系列趋化因子与干祖细胞相互作用[12]–[14]。本实验设计用半致死剂量^60^Co γ射线全身照射造成小鼠造血龛损伤，然后经尾静脉输注来自表达GFP荧光蛋白小鼠的洗涤血小板，在处理后第3天应用免疫荧光技术在小鼠的肝、脾、肺以及骨髓腔中观察到GFP荧光血小板，尤其是在骨髓中可以观察到更多GFP荧光血小板，表明血小板可以迁移到骨髓腔，为后续研究提供证据。

HSC具有自我更新和增殖分化的能力，能形成与造血相关的所有细胞，HPC可分为各系造血祖细胞，是各种造血细胞的前体细胞。在照射损伤模型中，输注组小鼠骨髓细胞计数在第3、7、14和21天均高于单纯照射组，外周血细胞计数也更高，其中第7天的血小板计数和第21天的白细胞计数差异有统计学意义，说明输注血小板有利于照射小鼠外周血象的恢复和提高骨髓细胞计数。在随后的流式细胞术检测中，我们观察到输注组在第7天和21天骨髓细胞增殖率更高，凋亡率更低，提示其发挥作用与促进增殖抑制凋亡相关。在移植模型中，通过28 d的观察周期，虽然两组外周血象的差别不大，这与复杂的造血机制相关，但是骨髓干祖细胞的比例有较大差别，BMT+PLT组均高于BMT组，且在第7天和28天表现为骨髓细胞增殖率更高，第14天凋亡率更低，骨髓细胞计数在第7和28天均多于BMT组，且差异有统计学意义。在移植后第7天，BMT+PLT组嵌合率高于BMT组，且较快达到90％。表明移植联合输注血小板可以促进HSC的归巢植入。

骨髓造血龛是HSC赖以生存的场所，其完整性是保证HSC发挥造血功能的首要条件，然而在移植前照射造成造血龛严重受损，影响了HSC的植入以及造血功能的发挥。有研究表明血小板是参与并调节体内受损血管的重要介质[15]，本实验经VE-Cadherin抗体的免疫荧光和免疫组化结果也表明，输注血小板后小鼠骨髓造血龛的完整性和连续性更好。

骨髓移植成功与否是多系统共同参与的过程，HSC的成功植入对提高移植成功率和效率来说至关重要[16]。受损的骨髓造血龛会影响HSC的归巢、定植以及造血重建。深入研究血小板在HSC的植入甚至造血龛修复中的作用机制，对于探寻新的有效的减轻骨髓造血龛损伤，加速HSC的植入，确保移植的成功具有重要的临床现实意义，为促进移植效率进一步提高，在治疗恶性血液病方面提供更加广阔的思路。
